# Machine Learning Metabolomics Profiling of Dietary Interventions from a Six-Week Randomised Trial

**DOI:** 10.3390/metabo14060311

**Published:** 2024-05-29

**Authors:** Afroditi Kouraki, Ana Nogal, Weronika Nocun, Panayiotis Louca, Amrita Vijay, Kari Wong, Gregory A. Michelotti, Cristina Menni, Ana M. Valdes

**Affiliations:** 1 Academic Unit of Injury, Recovery and Inflammation Sciences, Rheumatology, School of Medicine, University of Nottingham, Nottingham NG7 2UH, UK; 2 NIHR Nottingham Biomedical Research Centre, Nottingham University Hospitals NHS Trust and the University of Nottingham, Nottingham NG7 2UH, UK; 3 Department of Twin Research and Genetic Epidemiology, King’s College London, London SE1 7EH, UK; 4 Department of Epidemiology, Harvard T.H. Chan School of Public Health, Boston, MA 02115, USA; 5 Human Nutrition and Exercise Research Centre, Population Health Sciences Institute, Newcastle University, Newcastle upon Tyne NE2 4HH, UK; 6 Metabolon Inc., Research Triangle Park, Morrisville, NC 27560, USA; 7 Pain Centre Versus Arthritis, University of Nottingham, Nottingham NG5 1PB, UK

**Keywords:** fibre, omega-3, metabolomics, machine learning, indoleproprionate, gut microbiome

## Abstract

Metabolomics can uncover physiological responses to prebiotic fibre and omega-3 fatty acid supplements with known health benefits and identify response-specific metabolites. We profiled 534 stool and 799 serum metabolites in 64 healthy adults following a 6-week randomised trial comparing daily omega-3 versus inulin supplementation. Elastic net regressions were used to separately identify the serum and stool metabolites whose change in concentration discriminated between the two types of supplementations. Random forest was used to explore the gut microbiome’s contribution to the levels of the identified metabolites from matching stool samples. Changes in serum 3-carboxy-4-methyl-5-propyl-2-furanpropanoate and indoleproprionate levels accurately discriminated between fibre and omega-3 (area under the curve (AUC) = 0.87 [95% confidence interval (CI): 0.63–0.99]), while stool eicosapentaenoate indicated omega-3 supplementation (AUC = 0.86 [95% CI: 0.64–0.98]). Univariate analysis also showed significant increases in indoleproprionate with fibre, 3-carboxy-4-methyl-5-propyl-2-furanpropanoate, and eicosapentaenoate with omega-3. Out of these, only the change in indoleproprionate was partly explained by changes in the gut microbiome composition (AUC = 0.61 [95% CI: 0.58–0.64] and Rho = 0.21 [95% CI: 0.08–0.34]) and positively correlated with the increase in the abundance of the genus *Coprococcus* (*p* = 0.005). Changes in three metabolites discriminated between fibre and omega-3 supplementation. The increase in indoleproprionate with fibre was partly explained by shifts in the gut microbiome, particularly *Coprococcus*, previously linked to better health.

## 1. Introduction

Nutritional supplementation provides a convenient and effective means to address specific dietary deficiencies, optimise nutrient intake, and support overall health, ensuring individuals have access to a well-rounded spectrum of essential vitamins, minerals, and bioactive compounds for enhanced well-being [1].

A number of nutritional supplements are widely available which claim to reduce inflammation [2]. However, the mechanisms by which many supplements act are poorly understood [3], which may hinder the development of evidence-based nutritional recommendations and the ability of dietitians and clinicians to promote health and prevent disease through targeted nutrition.

Understanding the specific small molecules or metabolites altered by a nutritional supplement during controlled interventions can help provide valuable insights into the mechanistic pathways through which the supplement influences biochemical processes within the body, elucidating the molecular basis of its effects. Moreover, identifying altered metabolites offers potential biomarkers that can be used to monitor the efficacy and individual responsiveness to the supplement. Consequently, these biomarkers could serve as screening tools in future trials and provide information on the interplay between diet and metabolism, potentially informing personalized nutrition strategies that align with an individual’s specific metabolic profile [4].

Omega-3 polyunsaturated fatty acids are essential nutrients for humans and are a promising nutritional therapeutic for many disorders, as demonstrated in a vast array of studies [5], showing efficacy on improving cardiometabolic and mental health outcomes [6,7] via the effects of these fatty acids on reducing systemic inflammation [8].

Inulin is a form of dietary fibre classified as a fructan (a carbohydrate composed of fructose molecules linked together by glycosidic bonds) [9]. As a prebiotic fibre, inulin serves as a substrate for beneficial bacteria in the colon, encouraging their growth and activity. This fermentation process produces short-chain fatty acids (SCFAs) which have been widely documented [10]. Among its health benefits are reductions in inflammation and improvements in cardiometabolic parameters [10]. Furthermore, inulin shows potential for the improvement of various clinical outcomes. For example, inulin demonstrated potential for improving the glycaemic index in individuals with type 2 diabetes [11,12] and reducing serum uric acid levels, which are independently linked to higher mortality in renal failure patients [13].

We have previously reported the effects of both omega-3 (500 mg/day) and inulin fibre (20 g/day) supplementation on SCFAs and inflammatory markers [14]. However, a systematic analysis of the stool and circulating metabolites altered by these interventions has not yet been performed. Such characterisation could enhance our understanding of the underlying metabolic responses to these supplements and enable us to identify discriminating panels of metabolites that may contribute to their potential clinical benefits. In this study, we performed a secondary analysis of a two-arm nutritional supplementation intervention study that was designed to compare the effects of omega-3 and inulin fibre in healthy middle-aged and elderly volunteers over a six-week period. We used a large and broad commercial metabolite panel to investigate how changes in serum and stool metabolites map to the two different types of supplements and used a machine learning approach to identify the metabolites with discriminative value between the two types of nutritional supplementation.

## 2. Materials and Methods

### 2.1. Study Aim and Design

**Figure 1:**
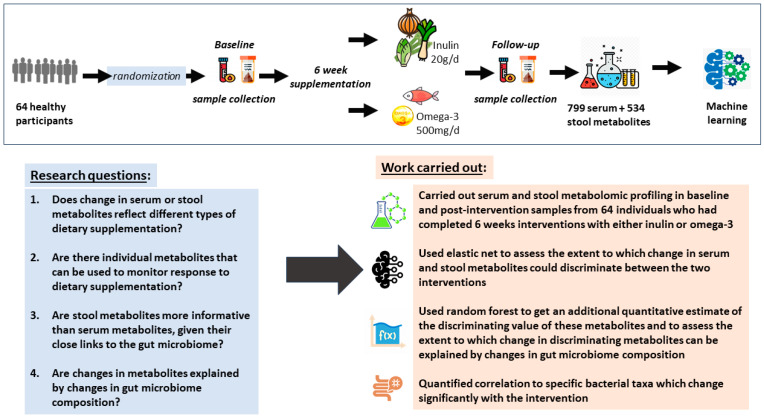
Schematic diagram of the study design.

We aim to uncover the physiological metabolic responses to daily omega-3 vs. inulin supplementation and identify key metabolites that are response specific. The study design and the specific research questions are presented in Figure 1.

### 2.2. Study Population

We included 64 participants from the TwinsUK registry who participated in the omega-3 and fibre intervention study and had concurrent stool and serum samples at baseline and follow-up. The omega-3 and fibre intervention study was a 6-week parallel randomised intervention trial designed to explore the influence of dietary omega-3 or fibre supplementation on gut microbial composition, as previously described [14]. Participants were randomised to one of two intervention arms, the first arm were administered 20 g of oral inulin fibre, while the second arm were given 500 mg of omega-3 supplements (165 mg of eicosapentaenoic acid (EPA), 110 mg docosahexaenoic acid (DHA) in gelatin capsules) daily for 6 weeks.

Patient selection, inclusion and exclusion criteria for the original intervention study have been described in detail previously [14]. Here, we conducted secondary analysis of the data from the original intervention study. Briefly, study subjects were enrolled from the TwinsUK registry, a national register of adult twins recruited as volunteers without selecting for any particular disease or traits. The TwinsUK cohort has been shown to be representative of the general population [15]. A total of 69 subjects were enrolled into the study and randomized into either the omega 3 or fiber arm. Participant eligibility included those aged >18 y who had a body mass index (BMI) between 20 and 39.9 kg/m^2^ and had a low habitual fiber consumption of less than 15 g/d. The following exclusion criteria were considered: ongoing or planned regular use of other omega-3 polyunsaturated fatty acid or cod liver oil supplements; seafood allergy; concomitant use of non-steroidal anti-inflammatory medications, including aspirin; current treatment for any chronic inflammatory condition or malignancy; previous colonic or small bowel resection; current smoker (minimum 6 months smoking cessation) and pregnancy. Clinical visits were conducted at the clinical research facility at St Thomas’ Hospital, London, UK. For the current secondary analysis, we were missing 5 aliquots/samples which were excluded from analysis. A detailed CONSORT flow diagram is included in Supplementary Figure S1.

Randomization was not stratified by gender and age and was performed using an online software (www.sealedenvelope.co.uk, accessed on 1 April 2018). Neither participants nor researchers were blinded to the interventions and hence allocation order as we previously reported [14]. Generation of a randomization schedule included obtaining the random numbers and assigning random numbers to each subject under the specific treatment conditions (conducted by Amrita Vijay). Participants were equally allocated between treatment arms (allocation ratio 1:1) with at least *n* > 32 in each arm. A power calculation was performed for the original intervention study as previously detailed in [14]. No changes to methods were made after trial commencement. In the intervention study data we analyzed, the majority of participants were singletons (*n* = 42). Eleven pairs of twins (*n* = 22) were included in the analysis, all of whom were assigned to different intervention groups.

### 2.3. Sample Collection

At the baseline and follow-up visits, anthropometric measurements (weight, height), blood, and stool were collected. During each visit, blood samples were collected from participants between 8:30 and 10 a.m. It was requested of the participants to arrive fasting, at least from 9 p.m. the previous evening. Serum Separator Tubes were used to collect blood samples, which were processed to separate serum and then aliquoted for storage at −80 °C within 2–3 h after collection. During research visits, stool samples were provided, and they were frozen at −80 °C until DNA extraction took place as soon as the study was over.

### 2.4. Metabolite Profiling

Liquid chromatography coupled with tandem mass spectrometry (LC-MS/MS) was performed by Metabolon Inc on serum and stool samples at two time points as previously described [16,17,18]. Full details and quality control have been described previously [17,18,19].

### 2.5. Microbiota Analysis

The method for stool DNA extraction has been reported in detail elsewhere [20]. Briefly, 100 mg of the sample were used for extraction without homogenization prior to this step. Stool samples were collected and the composition of the gut microbiome was determined by 16 S rRNA gene sequencing carried out as previously described [21].

In short, universal primers 355 F (CCAGACTCCTACGGGAGGCAGC) and 806 R (GGACTACHVGGGTWTCTAAT) and were used to amplify the V4 region of the 16S rRNA gene. MiSeq (Illumina Inc., San Diego, CA, USA) was used to sequence amplified DNA. The MYcrobiota pipeline was used to perform read filtering and clustering [22]. On the basis of a 97% similarity, reads were grouped into operational taxonomic units (OTUs) using closed-reference clustering against the SILVA database v132 after chimera sequences were filtered using Mothur’s VSEARCH algorithm. The OTU table was averaged after being rarefied 50 times to 7000 sequences per sample. These analyses were carried out in QIIME 2 (v2018.11).

### 2.6. Data Analysis

Statistical analysis was conducted using R (version 4.3.2), while we used GraphPad PRISM 10 for the illustrations.

#### 2.6.1. Metabolite QC

We included serum and stool metabolites present in at least 20% of the sample, which were day median normalised and inverse normalised. Missing values were imputed to the minimum.

#### 2.6.2. Elastic Net for Identifying Changes in Metabolites Reflecting Supplementation with Inulin or Omega-3

Elastic net models with five-fold cross-validation and repeated 5 times were performed separately on the serum and stool metabolites to determine if participants on fibre supplementation could be discriminated from participants on the omega-3 based on the changes in their serum and stool metabolite levels following supplementation. Covariates, age, sex, and BMI were included as independent variables in the model to assess whether they would influence the performance of the model. Repeated cross-validation was performed to identify the optimal model, based on an 80/20 random split into training and testing folds. Area under the curve (AUC) of the receiver operating characteristic (ROC) curve was used to select the optimal model using the largest value. Paired *t*-tests run separately for the fibre and omega-3 arms were used to illustrate at a univariate level the extent to which the metabolites identified changed with the dietary interventions. The most discriminating metabolites were selected based on whether they had non-zero regression coefficient from the elastic net.

#### 2.6.3. Sensitivity Analyses and Cross-Validation Using Random Forest and Logistic Regression

Sensitivity analyses and cross-validation were conducted on the elastic net results to assess by other methods the discriminating power of the metabolites identified in the previous section. We did this using Random forest classification and logistic regression models as described below, thus obtaining a distribution of AUC and Spearman’s Rho values on cross-validated data.

Logistic regression: The change in the most important metabolites was modelled using logistic regression with 5-fold cross validation and repeated 100 times to obtain robust estimates of the predictive performance of the models. Age, sex, and BMI were included as covariates and the base logistic regression model with only the covariates was computed to determine their significance. The data were randomly split into training and test sets (80/20). Mean AUC values across the 100 repeats and 5 folds were calculated to compare the performance of the elastic net models to the logistic regression models and *p*-values were computed for each metabolite to verify their significance.

Random forest (RF) classification: The change in the most important metabolites were modelled with 5-fold cross validated RF classification repeated 3 times to get an additional quantitative estimate of the discriminating value of these metabolites (randomForest function in R). The dataset was split into training set and testing set (80/20 random split). In the training set, recursive feature elimination was performed to identify the best number of features (i.e., the lowest number of features with the lowest error rate), and adaptive resampling used to tune mtry (number of predictors to sample at each split) and min_node_size (number of observations needed to keep splitting nodes). The mean AUC and the Spearman’s Rho (similarity between predicted and real values) across the cross validated repeated folds were calculated to compare the performance to the elastic net models and to get a measure of the strength of the association between the change in metabolites and supplementation.

#### 2.6.4. Associations of Differentiating Metabolites with SCFAs and Gut Microbiome Composition

RF regression and classification: In order to identify how much of the change in the levels of the most discriminating metabolites is explained by the changes in the gut microbiome we implemented RF regression and classification. The gut microbiome data were inverse normalised and predictors with variance zero or near zero were removed. A 5-fold cross-validation approach was implemented, based on an 80/20 random split into training and testing folds. The randomForest function in R was applied for both regressors (with parameters ntree = 1000 and mtry = one-third of the features number) and classifiers (with parameters ntree = 1000 and mtry = the square root of the features number). Performance was assessed using the mean of the Spearman’s and Pearson’s correlations between the actual and predicted metabolite levels across the 5 folds used as a test set. Change in metabolite levels was transformed into two classes by splitting the variable in two, and their performance was measured by calculating the mean of the AUC values obtained over the 5 folds. In secondary analysis, we also investigated whether baseline gut microbiome composition could predict changes in metabolites levels.

Correlations with SCFAs and specific genera: We then aimed to explore correlations of the most discriminating metabolites with SCFAs and specific genera previously identified to increase with the intervention [14]. To that end, we performed Spearman’s correlations between the changes in SCFAs and changes in *Coprococcus* and *Bifidobacterium* genera with changes in the most discriminating metabolites following the intervention.

## 3. Results

### 3.1. Descriptive Characteristics of the Participants

We included 64 healthy participants with serum and stool metabolomics profiling, who completed the 6 weeks intervention with either inulin or omega-3 and provided a sample at baseline and following the intervention (Figure 1). Half of the participants received inulin and the remaining half received omega-3. The mean age was 66.4 [standard error (SE): 1.2] years, the mean BMI was 26.6 (SE: 0.5) kg/m^2^, and 91% were female.

Due to the previously demonstrated lack of a correlation between the stool and blood metabolome [23], we independently examined the associations between changes in the levels of paired serum and stool metabolites with type of supplementation.

### 3.2. Changes in Serum Metabolites Differentiating between Inulin and Omega-3 Supplementation

**Figure 2:**
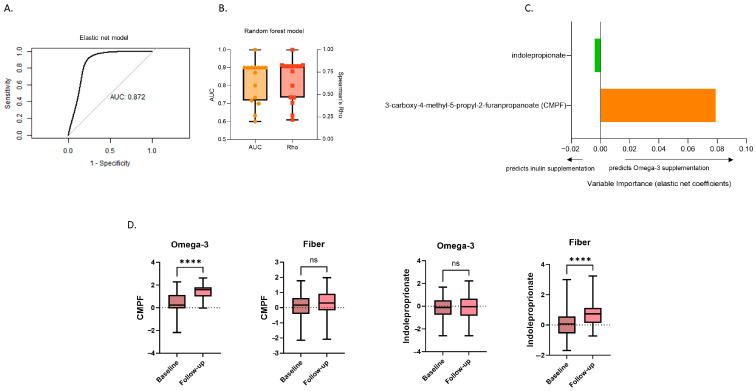
Changes in serum metabolomic data in response to 6 week supplementation with inulin or omega-3. (**A**) Receiver operating characteristic (ROC) curve using the selected features from the five-fold cross-validated elastic net model repeated five times; (**B**) boxplots of the performance [area under the curve (AUC) and quantitative estimate (Spearman’s correlation coefficient)] of a random forest model incorporating the metabolites identified by elastic net. The mean and 95% confidence intervals (CIs) of both the AUC and the Spearman’s Rho across the repeated folds are shown; (**C**) variance importance plot of the elastic net coefficients of the metabolites whose changed levels in serum were identified by elastic net regression as discriminating between the two interventions; and (**D**) boxplots showing the change in the identified serum metabolites in both arms separately (univariate paired *t*-test). ns: not significant, **** *p* < 0.0001.

Elastic net regression: We find that changes in circulating levels of 3-carboxy-4-methyl-5-propyl-2-furanpropanoate (CMPF) and indolepropionate (IPA), following dietary supplementation can discriminate between inulin and omega-3 supplementation with an AUC of 0.87 [95% confidence intervals (CI): 0.63–0.99] (Figure 2A). The variance importance plot indicates that an increase in CMPF circulating levels is reflective of supplementation with omega-3, while an increase in the levels of IPA predict supplementation with inulin fibre (Figure 2C). Consistent with the elastic net results, in univariate analysis, we find that levels of CMPF significantly increased from baseline to follow-up only in the omega-3 group (*p* = 1.48 × 10^−8^) whereas levels of IPA increased only following fibre supplementation (*p* = 1.90 × 10^−4^) (Figure 2D).

Random forest and logistic regression: The differentiating metabolites, IPA and CMPF, were included in cross-validated repeated logistic regression and random forest to further validate their discriminating capacity in sensitivity analyses. The AUCs and Spearman’s Rho from repeated cross-validated random forest classification are presented in Figure 2B. Consistent with the performance of the elastic net model, the mean AUCs from the logistic and random forest models were 0.82 [standard deviation (SD): 0.11] and 0.76 (SD: 0.09), respectively, indicating a successful separation between the omega-3 and fibre groups. Adjusting the logistic and elastic net regression models for selected covariates, namely age, sex, and body mass index, did not alter their performance, and the covariates themselves were not found to be statistically significant in the base logistic regression model (Supplementary Table S1, Supplementary Materials). The random forest mean Spearman’s Rho of 0.54 (SD: 0.18) indicates a strong association between changes in the two discriminating metabolites and the type of supplementation (please refer to Supplementary Table S2 in Supplementary Materials for full sensitivity analysis results).

### 3.3. Changes in Stool Metabolites Differentiating between Inulin and Omega-3 Supplementation

**Figure 3:**
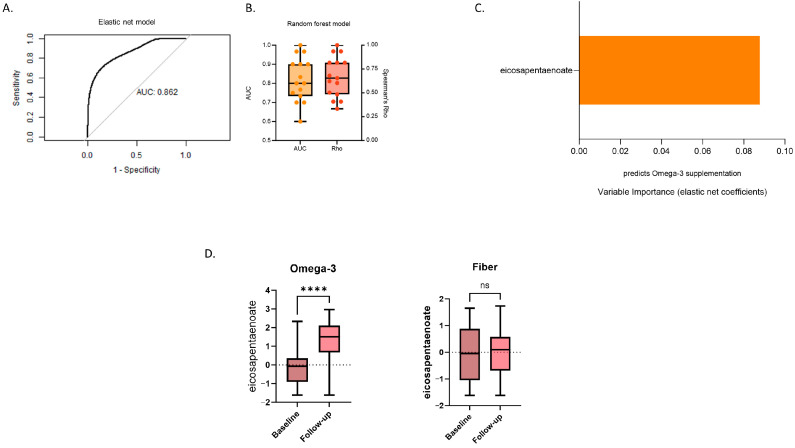
Changes in stool metabolomic data in response to 6 week supplementation with inulin or omega-3. (**A**) ROC curve using the selected feature from the five-fold cross-validated elastic net model repeated five times; (**B**) boxplots of the performance (AUC) and quantitative estimate (Spearman’s correlation coefficient) of a random forest model incorporating the metabolites identified by elastic net. The mean and 95% CIs of both the AUC and the Spearman’s Rho across the repeated folds are shown; (**C**) variance importance plot (elastic net coefficient) of the metabolites whose changed levels in serum were identified by elastic net regression as discriminating between the two interventions; and (**D**) boxplots showing the change in the identified serum metabolites in both arms (univariate paired *t*-test). ns: not significant, **** *p* < 0.0001.

Elastic net regression: Among the stool metabolites, eicosapentaenoate (EPA) was the single and top discriminating variable between fiber and omega-3 supplementation with high accuracy (elastic net regression AUC = 0.86 [95% CI: 0.64–0.98]) (Figure 3A). Changes in the level of this stool metabolite following the intervention were reflective of supplementation with omega-3 as shown from Figure 3C. Moreover, in line with this result, we find that EPA significantly increased from baseline to follow-up only in the Omega-3 group (*p* = 1.69 × 10^−7^) (Figure 3D). None of the stool metabolites were associated with fibre supplementation.

Random forest and logistic regression: We also performed sensitivity analyses using cross-validated repeated logistic regression and random forest models to further evaluate the discriminating capability of EPA. The AUCs and Spearman’s Rho from the cross-validated repeated random forest classification are illustrated in Figure 3B. Aligning with the elastic net model’s performance, the mean AUCs from the logistic and random forest models were 0.78 (SD: 0.12) and 0.82 (SD: 0.12), respectively. This suggests that variations in EPA levels following the intervention effectively differentiate between the omega-3 and inulin supplementation groups. Both logistic and elastic net models were adjusted to account for the selected covariates, which were not found to be statistically significant in logistic regression (Supplementary Table S1, Supplementary Materials) and did not alter the predictive performance of the models. Similar to the two discriminative serum metabolites, there was a robust association between changes in EPA and the type of supplementation as indicated by the random forest mean Spearman’s Rho of 0.67 (SD: 0.21) (please refer to Supplementary Table S2 in Supplementary Materials for the full sensitivity analysis results).

### 3.4. Associations of Differentiating Metabolites with SCFAs and Gut Microbiome Composition

Due to the known interplay between the gut microbiome with the serum and stool metabolome [24,25], we next explored whether changes in gut microbiome composition and its metabolites (SCFAs) can explain some of the variation in the levels of differentiating metabolites.

**Figure 4:**
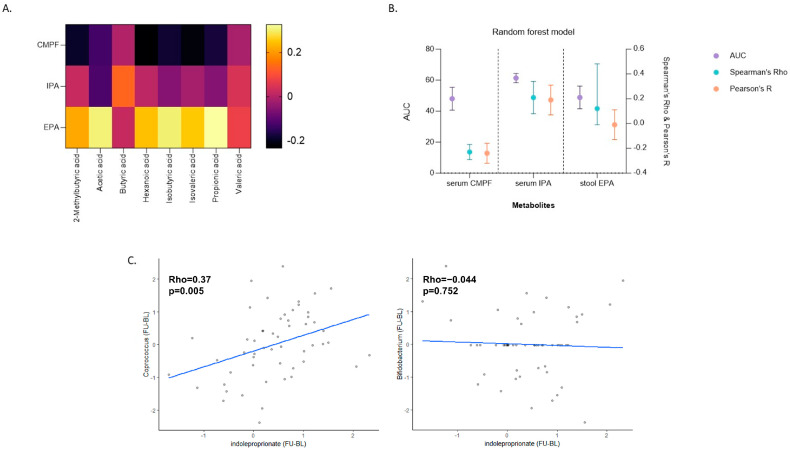
Associations of differentiating metabolites with SCFAs and gut microbiome composition. (**A**) Lack of association between the change in discriminating metabolites and change in circulating SCFA levels; (**B**) Random forest model showing to what extent can changes in gut microbiome composition in response to the intervention explain changes in the metabolites that can discriminate between the two interventions; and (**C**) Spearman’s correlations between change in circulating IPA and changes in two key taxa which increased with the intervention (*Coprococcus* and *Bifidobacterium*).

SCFAs: We explored associations between change in the differentiating serum and stool metabolites, CMPF, IPA and EPA, with changes in SCFAs following the intervention. No associations were found to be significant after adjusting for multiple testing (FDR < 0.05) (Figure 4A).

Gut microbiome composition: RF classification and regression analysis revealed that out of the three differentiating metabolites, only change in IPA was sufficiently explained (AUC > 60%) by changes in the gut microbiome (AUC = 0.61 [95% CI = 0.58–0.64] and Spearman’s Rho = 0.21 [95% CI = 0.08–0.34]). Changes in CMPF and EPA were not sufficiently explained by changes in the gut microbiome (Figure 4B). Furthermore, we conducted RF to explore whether the baseline gut microbiome could predict baseline levels of CMPF, IPA, and EPA, as well as changes in these metabolites following the intervention (Supplementary Table S3, Supplementary Materials). Our findings indicate that the baseline gut microbiome has a minimal predictive effect on changes in IPA, while it can sufficiently predict baseline levels of both serum metabolites.

Gut microbiome and IPA: Lastly, to illustrate the links between IPA and SCFA producers we show IPA’s association with two SCFA-producing genera, *Bifidobacterium* and *Coprococcus*, which we previously reported to increase with the intervention [14]. We focused on the taxa *Coprococcus* and *Bifidobacterium* as these were also classed as the first and second most important variables (taxa) from the random forest model partly explaining variation in change in IPA. Out of the two, only change in *Coprococcus* was positively correlated with a change in IPA (*p* = 0.005). (Figure 4C).

## 4. Discussion

In the first study to compare changes in serum and stool metabolomics profiles following dietary supplementation with inulin and omega-3, we find two serum and one stool metabolites that reliably distinguish between inulin (fiber) and omega-3 supplementation, respectively. Specifically, we find that (i) fibre supplementation is accurately reflected by elevated IPA in serum, while omega-3 supplementation is reflected by increased serum CMPF, (ii) higher levels of stool EPA accurately indicate supplementation with omega-3, whereas no stool metabolites reflect supplementation with fibre, and (iii) the increase in IPA is partly explained by shifts in the whole of the gut microbiome, and specifically by an increase in the genus *Coprococcus*, whereas the increases in CMPF and EPA indicative of supplementation with omega-3 cannot be reliably explained by the changes in the gut microbiome.

Wide-scale metabolomics analysis integrated with other omics including the gut microbiome and machine learning broadens the possibility of novel discoveries and can validate previous findings. Inulin appears to act via the tryptophan metabolism pathway apart from its well-established SCFA mechanism of action [10] whereas omega-3 seems to exert its effect via the production of resolvins and protectins but also by changing the membrane composition of cells which affects production proinflammatory peptide mediators such as cytokines or adhesion molecules.

IPA: The finding that supplementation with inulin can be accurately predicted by increased IPA in serum confirms previous findings from observational studies that observed a significant link between IPA and both dietary total carbohydrate and fibre intakes [26,27,28,29]. IPA is a tryptophan derivative of the microbiota pathway [30] and is associated with reduced likelihood of type 2 diabetes [26]. Furthermore, IPA might play a role in the pathophysiology of liver and cardiovascular disease as it was found to be decreased in alcoholic hepatitis and cirrhosis patients compared to controls [31] and was linked to advanced cardiovascular disease over and above traditional atherosclerosis risk factors [32]. Our results showed for the first time that changes in IPA following fibre supplementation are explained by shifts in the gut microbiome whereas baseline gut microbiome was minimally predictive of changes in IPA. Consistent with our previous work [27], baseline levels of IPA were substantially explained by the baseline gut microbiome reinforcing the idea that IPA is correlated with the gut microbiome as a whole. Here we also show that supplementation with inulin for 6-weeks can induce changes in the levels of serum IPA coupled by changes in the gut microbiome which may offer actionable targets easily modifiable through diet interventions for the prevention of various metabolic diseases. Furthermore, we show that an increase in IPA was strongly correlated with an increase in *Coprococcus* which agrees with previous findings from our group [14,27]. None of the strains previously identified in vitro as IPA producers mainly *Peptostreptococcus* spp. and *Clostridium* spp. [33] showed correlation with IPA most likely due to the limited resolution of the 16S data. However, we do find an increase in a genus within the *Clostridiales* order, commonly associated with IPA production, showed a strong correlation with elevated IPA levels.

CMPF: Elevated CMPF in serum strongly and accurately reflects omega-3 supplementation. This aligns with previous findings from a targeted metabolomic analysis in a cohort of 7 to 12 healthy volunteers using higher omega-3 doses [34]. Our untargeted metabolomics approach on a cohort of 64 healthy adults validates this association at a much lower dose of 165 mg EPA and 110 mg DHA. When mice were given CMPF before or after being fed a high-fat diet, it enhanced overall lipid metabolism, improved insulin sensitivity, increased beta-oxidation, lowered lipogenic gene expression, and mitigated steatosis [34]. In humans, patients with type 2 diabetes serum CMPF exhibited an inverse correlation with serum triglycerides, a risk factor for diabetes and cardiovascular diseases [35,36]. Furthermore, a recent Mendelian Randomisation study found that CMPF was associated with a lower risk of sepsis [37] and reduced risk of infection in childhood [38]. Taken together these results suggest that CMPF might play a role in the enhanced metabolic and other health benefits associated with omega-3 supplementation.

EPA: The variation in EPA levels in faeces, predicting omega-3 supplementation, may reflect levels of unabsorbed EPA, the primary ingredient of the omega-3 supplement [39,40]. It appears that circulating serum metabolites, more directly linked to metabolic health [14], are effective at elucidating differences in metabolic pathways between the two types of supplements.

There was a lack of association between changes in circulating SCFAs and alterations in the distinctive metabolites—IPA, CMPF, and EPA—and between the change in IPA and the change in *Bifidobacterium*. This lack of association is unsurprising, as although these metabolites can have similar effects on the host, they operate through different mechanisms. For instance, even though both IPA and acetate can be produced by *Clostridium* spp., they follow distinct pathways [41]. Additionally, *Bifidobacterium* appears to be engaged in pathways related to IPA, but this effect does not seem to be mediated by fibre [29] and while there no evidence that it can produce IPA, it is suggested to be able to produce IPA’s substrate, indolelactate [29].

There are several strengths with this study. These include the extensive metabolite panels and machine learning techniques used, the interventional nature of the study and the incorporation of multi-omics, namely metabolomics and the gut microbiome. In addition to these strengths, it is important to highlight the novelty of the present study compared to our prior research that focused on inflammatory cytokines and cardiovascular risk factors in response to either inulin or omega-3 intervention [14], or solely examined IPA cross-sectionally [27]. Here instead, we have comprehensively investigated a wide range of serum and fecal small molecules in response to these interventions. Furthermore, prior investigations were targeted or observational and examined these interventions in isolation as highlighted throughout this Discussion. In contrast, our study used an untargeted approach within the same randomized controlled trial, uncovering differences in metabolite profiles following supplementation with either inulin or omega-3. This design is optimal for identifying variations in metabolic responses to these supplements, potentially serving as biomarkers for comparing efficacy and individual responsiveness.

We also note some limitations. First, although we used cross-validation and we validated our results using different statistical methods, the study lacked an independent validation cohort, which would have further reinforced our results. However, our results are consistent with previous findings, highlighting the robustness of our approach. Second, we used 16s RNA sequencing data. Shotgun metagenomic data would have added more in-depth information about the species and strains related to the differentiating metabolites identified in our study. Third, we acknowledge that a larger sample size could potentially reveal additional metabolite-intervention relationships. However, we performed elastic net regression as it has been demonstrated to perform well and achieve high predictive accuracy in situations where there are many predictors relative to the sample size [42]. In addition, we conducted post-hoc power analysis for each metabolite to further validate that our results are not false positives: for IPA, where the mean was 0.73 (SD = 0.83) for inulin and 0.003 (SD = 1.08) for omega-3, we achieved 85% power to detect differences between the two groups at alpha level 0.05. For CMPF, with mean values of 0.27 (SD = 1.01) for inulin and 1.46 (SD = 0.58) omega-3, we achieved 99% power to detect differences between the two groups at alpha level 0.001. Similarly, for EPA, with a mean of 0.10 (SD = 0.83) for inulin and 1.43 (SD = 0.97) omega-3, we obtained 99% power to detect differences between the two groups at alpha level 0.001. Fourth, we acknowledge that an AUC = 0.61 [95% CI: 0.58–0.64] and Rho = 0.21 [95% CI: 0.08–0.34] do not provide a solid basis for prediction. However, with regards to the study of the gut microbiome, our primary focus was to explore whether changes in its composition as a whole explained some of the variation in the change in the levels of discriminating metabolites. As the AUC is above 0.60 (i.e., the prediction is not completely at random) and Rho = 0.21, we can infer that the gut microbiome is correlated with the metabolite even weakly, and that shifts in gut microbiome composition partly explain some of the variation in the change in the level of IPA. Finally, the dietary intervention was conducted in healthy volunteers. Given the role of these supplements and their metabolite signatures in metabolic health, future wide-scale metabolomic analysis of intervention studies in diseased cohorts would verify the beneficial roles of these supplements and their metabolic pathways of action.

## 5. Conclusions

These findings contribute to our understanding of the differential host metabolic responses to inulin and omega-3. Two key serum metabolites were reflective of which type of dietary intervention was performed, IPA for inulin and CMPF for omega-3. This insight may shed light on the mechanisms underlying the relevance of these metabolites to human metabolic health and disease as well as provide practical, targeted dietary interventions tailored to individuals based on their circulating metabolite profiles.

## Data Availability

The metabolomics data used in this study are held by the Department of Twin Research at King’s College London and are only accessible via managed access due to a contractual agreement between the Department of Twin Research at King’s College London and Metabolon inc. These data can be released to bona fide researchers using our normal procedures overseen by the Wellcome Trust and its guidelines as part of their core funding (https://twinsuk.ac.uk/resources-for-researchers/access-our-data/, accessed on 26 June 2023). The gut microbiome data are available on Mendeley (DOI: 10.17632/p48y674p9n.1, accessed on 12 April 2024). Any additional information required to reanalyze the data reported in this paper is available from the corresponding author upon request.

## Supplementary Materials

The following supporting information can be downloaded at: https://www.mdpi.com/article/10.3390/metabo14060311/s1, Figure S1. CONSORT diagram illustrating both the original intervention study and the samples used in the current secondary analyses, detailing the numbers of participants assigned randomly to each group, those who underwent interventions, and those included in the analysis for the outcomes of interest; Table S1: Results from the base logistic regression model including only the selected covariates, age, sex, and body mass index (BMI); Table S2: Sensitivity analysis to validate the serum and stool metabolites with predictive value for discrimination between the omega-3 and inulin fiber nutritional supplementation by 5-fold cross validated logistic regression models repeated 100 times (using AUC and *p*-values) and 5-fold cross validated Random Forest classification (using AUC and Spearman’s correlations) models repeated 3 times adjusted for age, sex and body mass index. The mean AUC and the Spearman’s Rho across the repeated folds are shown; Table S3: Influence of the baseline gut microbiota composition in baseline levels of serum and faecal metabolites, as well as changes in these metabolites estimated by Random Forest regression (using Spearman’s correlations). The mean values and the 95% confidence intervals of the Spearman’s correlation between the real value of each component and the value predicted by regression models across training/testing folds are shown.
