# Supplementary material for: Machine Learning Metabolomics Profiling of Dietary Interventions from a Six-Week Randomised Trial
Source: Metabolites. 2024 May 29;14(6):311. doi: 10.3390/metabo14060311 (PMC11205626; doi:10.3390/metabo14060311)
Supplement: Supplementary file 1 [file metabolites-14-00311-s001.zip › metabolites-3008603-supplementary.pdf]

metabolites

MDPI

Supplementary Materials: Machine Learning Metabolomics Profiling of Dietary Interventions from a Six-Week Randomised Trial

Afroditi Kouraki [1,2,*], Ana Nogal [3,4], Weronika Nocun [1], Panayiotis Louca [3,5], Amrita Vijay [1], Kari Wong [6], Gregory A. Michelotti [6], Cristina Menni [3,†] and Ana M. Valdes [1,2,7,†]

1 Academic Unit of Injury, Recovery and Inflammation Sciences, Rheumatology, School of Medicine, University of Nottingham, Nottingham, NG7 2UH ,UK
2 NIHR Nottingham Biomedical Research Centre, Nottingham University Hospitals NHS Trust and the University of Nottingham, Nottingham, NG7 2UH, UK
3 Department of Twin Research and Genetic Epidemiology, King's College London, London, SE1 7EH, UK
4 Department of Epidemiology, Harvard T.H. Chan School of Public Health, Boston, MA, 02115, USA
5 Human Nutrition and Exercise Research Centre, Population Health Sciences Institute, Newcastle University, Newcastle upon Tyne NE2 4HH, UK
6 Metabolon Inc., Research Triangle Park, Morrisville, NC 27560, USA
7 Pain Centre Versus Arthritis, University of Nottingham, Nottingham, NG5 1PB, UK
* Correspondence: afroditi.kouraki1@nottingham.ac.uk
† These authors contributed equally to this work.

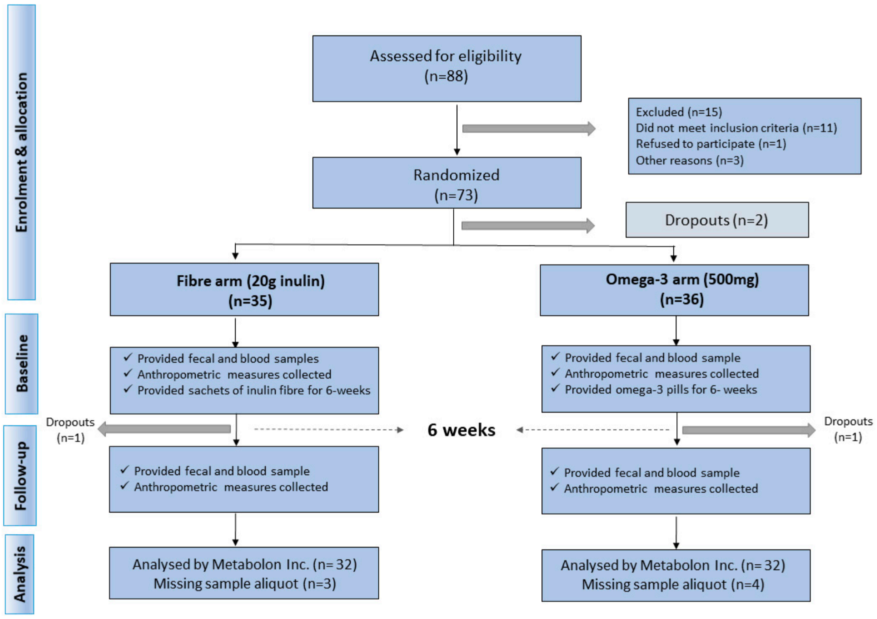

Figure S1. CONSORT diagram illustrating both the original intervention study and the samples used in the current secondary analyses, detailing the numbers of participants assigned randomly to each group, those who underwent interventions, and those included in the analysis for the outcomes of interest.

Table S1. Results from the base logistic regression model including only the selected covariates, age, sex, and body mass index (BMI).

Sample	*n*	Variables	Estimate (SE)	*p*-value
Serum	64	Age	−0.02 (0.03)	0.395
		Sex	−1.02 (0.95)	0.286
		BMI	−0.02 (0.06)	0.745
Stool	62	Age	−0.02 (0.03)	0.415
		Sex	−0.50 (0.90)	0.579
		BMI	−0.01 (0.06)	0.843

Table S2. Sensitivity analysis to validate the serum and stool metabolites with predictive value for discrimination between the omega-3 and inulin fiber nutritional supplementation by 5-fold cross validated logistic regression models repeated 100 times (using AUC and p-values) and 5-fold cross validated Random Forest classification (using AUC and Spearman's correlations) models repeated 3 times adjusted for age, sex and body mass index. The mean AUC and the Spearman's Rho across the repeated folds are shown.

Sample	Metabolite	Logistic regression Mean AUC (SD)	Logistic regression P-value	Random forest Mean AUC (SD)	Random forest Mean Spearman's Rho (SD)
Serum	CMPF and IPA	0.82 (0.11)	CMPF: 0.0002; IPA: 0.0043	0.76 (0.09)	0.54 (0.18)
Stool	EPA	0.78 (0.12)	0.0003	0.82 (0.12)	0.67 (0.21)

CMPF; 3-carboxy-4-methyl-5-propyl-2-furanpropanoate; IPA; indolepropionate; EPA; eicosapentaenoate.

Table S3. Influence of the baseline gut microbiota composition in baseline levels of serum and faecal metabolites, as well as changes in these metabolites estimated by Random Forest regression (using Spearman's correlations). The mean values and the 95% confidence intervals of the Spearman's correlation between the real value of each component and the value predicted by regression models across training/testing folds are shown.

Time-point	Sample	Metabolite	Spearman's Rho	Spearman's Rho 95% CI
Baseline	Serum	CMPF	−0.09	−0.19, 0.01
		IPA	0.05	0.02, 0.08
	Stool	EPA	−0.17	−0.24, −0.1
Change	Serum	CMPF	0.22	0.16, 0.28
		IPA	0.18	0.14, 0.24
	Stool	EPA	0.04	−0.03, 0.11

CMPF: 3-carboxy-4-methyl-5-propyl-2-furanpropanoate; IPA: indolepropionate; EPA: eicosapentaenoate.
